# White or Imperial Rhubarb

**Published:** 1853-11

**Authors:** G. Walpers


*White or Imperial Rhubarb.* By Dr. G. Walpers.—In all works on Pharmacology there occurs a somewhat vague account of a very superior kind of rhubarb, said to be collected for the sole and particular use of the Imperial Court of St. Petersburgh, and distinguished by the name of *White* or *Imperial Rhubarb* (*Radix Rhei alba seu imperialis.*) It is described as a rhubarb root in which the white portion so far predominates that only a few red streaks are perceptible upon the surface of a transverse section. No one, however, is from personal knowledge acquainted with this species of rhubarb. In order to put an end to these doubts, I some time since addressed a letter to Mr. Buchner, Chief Apothecary to the Imperial Court, begging him for a small specimen of this "*Imperial Rhubarb*" for my pharmacological collection, or should the communication of this precious drug be inadmissible, that I might at least have an authentic description of it. Mr. Buchner replied to this request with the utmost promptitude, informing me that, after having instituted the most careful inquiries, it appeared that no such species of rhubarb had at any time been imported for the Imperial family; that it had never occurred in commerce; and, finally, that in neither any public or private collection in St. Petersburgh was there to be found a specimen of this (consequently mythical) root.—*Bonplandia*, for March 15, 1853.

[It was the Russian traveller, Pallas, who first drew attention to the so-called *White Rhubarb.* We extract from his works the passage relating to it: "J'ai vu pendant mon sejour à Kiakta des petits morceaux de rhubarbe blancs comme du lait. Elle est douce au goût, et a les mêmes propriétés que celle de la meilleure qualité. L'apothecaire se proposoit de trier tous ces morceaux, et de les envoyer separément á Petersburg pour la pharmacie de la Cour.'—*Voyages de M. P. S. Pallas, en différentes, Provinces de l'Empire de Russie, et dans l'Asie Septentrionale, traduits de l' Allemand.* Paris, 4to, 1793, tome iv. p. 219.—Ed. *N. Y. Ph. Journ.*]

